# Differential Glycosite Profiling—A Versatile Method to Compare Membrane Glycoproteomes

**DOI:** 10.3390/molecules26123564

**Published:** 2021-06-10

**Authors:** Malwina Michalak, Martin Simon Kalteis, Aysel Ahadova, Matthias Kloor, Mark Kriegsmann, Katharina Kriegsmann, Uwe Warnken, Dominic Helm, Jürgen Kopitz

**Affiliations:** 1 Department of Applied Tumor Biology, Institute of Pathology, Heidelberg University Hospital, Im Neuenheimer Feld 224, 69120 Heidelberg, Germany; simon.kalteis@med.uni-heidelberg.de (M.S.K.); aysel.ahadova@med.uni-heidelberg.de (A.A.); matthias.kloor@med.uni-heidelberg.de (M.K.); 2 Clinical Cooperation Unit Applied Tumor Biology, DKFZ (German Cancer Research Center) Heidelberg, Im Neuenheimer Feld 280, 69120 Heidelberg, Germany; 3 Institute of Pathology, Heidelberg University Hospital, Im Neuenheimer Feld 224, 69120 Heidelberg, Germany; mark.kriegsmann@med.uni-heidelberg.de; 4 Department of Hematology, Oncology and Rheumatology, Heidelberg University Hospital, Im Neuenheimer Feld 410, 69120 Heidelberg, Germany; katharina.kriegsmann@med.uni-heidelberg.de; 5 Clinical Cooperation Unit Neurooncology, DKFZ (German Cancer Research Center), Im Neuenheimer Feld 280, 69120 Heidelberg, Germany; u.warnken@dkfz.de; 6 Genomics and Proteomics Core Facility, MS-based Protein Analysis Unit, DKFZ (German Cancer Research Center) Heidelberg, Im Neuenheimer Feld 280, 69120 Heidelberg, Germany; d.helm@dkfz.de

**Keywords:** glycoproteomics, glycosite, colorectal cancer, glycosylation, glycoprotein, mass spectrometry, glycopeptide

## Abstract

Glycosylation is the most prevalent and varied form of post-translational protein modifications. Protein glycosylation regulates multiple cellular functions, including protein folding, cell adhesion, molecular trafficking and clearance, receptor activation, signal transduction, and endocytosis. In particular, membrane proteins are frequently highly glycosylated, which is both linked to physiological processes and of high relevance in various disease mechanisms. The cellular glycome is increasingly considered to be a therapeutic target. Here we describe a new strategy to compare membrane glycoproteomes, thereby identifying proteins with altered glycan structures and the respective glycosites. The workflow started with an optimized procedure for the digestion of membrane proteins followed by the lectin-based isolation of glycopeptides. Since alterations in the glycan part of a glycopeptide cause mass alterations, analytical size exclusion chromatography was applied to detect these mass shifts. N-glycosidase treatment combined with nanoUPLC-coupled mass spectrometry identified the altered glycoproteins and respective glycosites. The methodology was established using the colon cancer cell line CX1, which was treated with 2-deoxy-glucose—a modulator of N-glycosylation. The described methodology is not restricted to cell culture, as it can also be adapted to tissue samples or body fluids. Altogether, it is a useful module in various experimental settings that target glycan functions.

## 1. Introduction

More than 50% of all proteins in humans and animals are glycosylated. Most soluble and membrane-bound proteins expressed in the endoplasmic reticulum are glycosylated to some extent, including secreted proteins, surface receptors and ligands, organelle-resident proteins, and (to some extent) cytoplasmic proteins. Through this high abundance and its tremendous variety, glycosylation increases the diversity of the proteome to a level unmatched by any other post-translational modification. This variety is brought about by the concerted action of a multitude of glycosyltransferases (GTs), glycosidases, and nucleotide sugar transporters that reside in the ER and Golgi and that function together to build well-defined saccharide modifications of proteins [1]. N- and O-glycosylation, as well as glypiation, are the most common types of glycosylation [2,3,4]. Though glypiation, i.e., the addition by covalent bonding of a glycosylphosphatidylinositol (GPI) anchor, is a common post-translational modification that localizes proteins to cell membranes, N- and O-glycosylation confers various physicochemical and biological functions to a protein. These physicochemical functions include (I) the modification of solubility, electrical charge, mass, size and viscosity in solution; (II) the control of protein folding; (III) the stabilization of conformation; (IV) the conferral of chemical stability; and (IV) protection against proteolysis [5]. Our knowledge on the biological functions of protein glycosylation is still only the tip of the iceberg. However, the huge effort that a cell makes to modify the majority of proteins with glycan structures clearly corroborates that this posttranslational modification is of key importance for cellular functioning [1]. Accordingly, recent advances in understanding specific functions of a cell’s glycome in health and disease have proven this concept. There is rapidly increasing evidence that glycan modifications can specifically affect the functions of proteins, thereby regulating cell adhesion [6], molecular trafficking and clearance [7,8], receptor activation [9], signal transduction [10,11], and endocytosis [12,13]. Altogether, defined glycosylation appears to be a prerequisite to the proper function of many proteins. Correspondingly, even slight changes of glycosylation can disrupt or alter normal cellular functions [14]. Hence, altered glycosylation is involved in a variety of different diseases, including cancer [15], neurological disorders [16,17], immune deficiencies and autoimmunity [18], and inflammation [19]. In particular, cancer-associated glycosylation changes have attracted broad interest, since these alterations are involved in different steps of tumour progression, i.e., tumour proliferation, invasiveness, metastasis, and angiogenesis [15]. Therefore, aberrant glycan structures are both applicable as valuable diagnostic markers [20] and considered to be novel therapeutic targets [21,22].

The huge significance of protein glycosylation for physiological and pathological cellular functions has led to the development of a large repertoire of analytical tools to analyse the structure of glycoproteins, ranging from simple staining methods [23] to sophisticated procedures based on mass spectrometry, NMR, and glycan arrays [24,25]. However, in addition to such structure-focused glycomics, methods to globally screen for changes in the glycoproteome as a response to physiological or pathological events with specific changes of protein glycan decoration are mandatory as a starter for investigations regarding how the glycoprotein modifications are involved in such alterations of the cellular state and behaviour. Such methods that compare the glycoproteome of different cellular states, thereby identifying proteins that contribute to these “event-related” glycoproteome changes, are, in contrast to structure-orientated methodologies, much less frequently described. Events that are able to induce glycoproteome changes are multifarious, e.g., growth and differentiation signals, gene mutations, and drug action. Metabolic labelling with isotopically labelled glycan building blocks or clickable sugar-derivatives has been applied to screen for such functional glycoproteome changes [26,27]. Other approaches combine the lectin- or antibody-based isolation of glycopeptides or glycoproteins with column chromatography and mass spectrometric (glyco)-proteomics [28]. 

Nevertheless, glycomics and the glycoproteomic analysis of glycoproteins are still challenging endeavours that require sophisticated instrumentation and specific expertise [29]. Accordingly, only a very limited number of labs have the expertise required to accomplish this task. On the other hand, glycobiology is a rapidly growing field of broad interest. Therefore, the intention of the present paper was to provide researchers that are engaged in the proteomic field but have no special expertise in glycoproteomics with a simple method to check whether alterations in protein N-glycosylation might play a role in their analytical work. Therefore, we describe a novel strategy to compare cellular glycoproteomes, thus identifying membrane proteins and the affected glycosite in these proteins. The workflow consisted of an optimized procedure for the digestion of membrane proteins followed by the lectin-based isolation of glycopeptides and their fractionation. Since alterations in the glycan part of a glycopeptide cause mass alterations, analytical size exclusion was applied to detect these mass shifts. A combination of N-glycosidase treatment, ^18^O-isotopic labelling, and LC–MS/MS mass spectrometry finally identified proteins with altered glycan structures and the respective glycosites. In order to establish and test the strategy, we treated the human colon cancer cell line CX1 with 2-deoxy-D-glucose (2-DG), which functions as a mannose-mimetic molecule and thereby acts as a modulator of N-glycosylation [30]. Initially, 2DG was developed as an anticancer agent. It accumulates in tumour cells due to the Warburg effect, where it interferes with glucose metabolism [31,32]. Since mannose is the C2 epimer of glucose, 2DG is equivalent to 2-deoxymannose. Accordingly, the incorporation of 2DG into GDP-2-DG competes with GDP-mannose for incorporation by mannosyltransferases into N-glycans, resulting in interference with the N-glycosylation of proteins [33].

## 2. Results

### 2.1. Optimization of Sample Preparation for LC–MS/MS

For the purpose of developing an efficient and easy-to-handle methodology for comparing N-glycoproteomes between two conditions, we worked out a strategy that is based on (I) the isolation of glycopeptide pools from both conditions and (II) the detection of differences between the pools that (III) allow for the identification of proteins with altered glycan structures and the respective glycosites by LC–MS/MS and bioinformatic tools. We firstly aimed to optimize sample preparation steps to prepare the glycopeptide pools while considering cost efficiency, time efficiency, robustness, and compatibility with the next steps of the procedure, including LC–MS/MS analysis. Human CX1 colon carcinoma cells were applied as a model in all experiments. 

Since N-glycosylation mostly occurs on membrane-bound cellular proteins [34], membrane preparations had to be used as the starting material, and membrane protein precipitates resulted from the initial extraction of the starting material. We aimed firstly to perform the efficient enzymatic digestion of a high amount (4 mg) of the protein precipitate obtained from the model cell line. While different digestion strategies were compared (see Figure S1 and Supplementary Materials Section 1.1), our study showed that for high amounts of precipitated protein in-solution, digestion in the presence of urea using two enzymes (Lys-C (for pre-digestion at 6M urea) and trypsin (for digestion at 1 M urea)) followed by stage tipping preparation for the subsequent glycopeptide enrichment was characterized by high efficiency, low costs, and robustness.

As a next step after the enzymatic protein digestion, we chose the appropriate glycopeptide enrichment strategy. Since we focused on protein N-glycosylation for method development, our glycopeptide enrichment targeted N-glycopeptides. Most eukaryote N-glycosylation can be characterized by a high presence of mannose residues [35]. For that reason, we chose mannose-binding plant lectin concanavalin A (ConA) [36] to capture N-glycosylated peptides. A comparison of two common solid phase extraction methods proved streptavidin-bound magnetic beads coupled with biotinylated ConA to be the better strategy for the enrichment of N-glycosylated peptide while ensuring the purity of the glycopeptide sample (see Figure S2 and Supplementary Materials Section 1.2).

**Figure 1:**
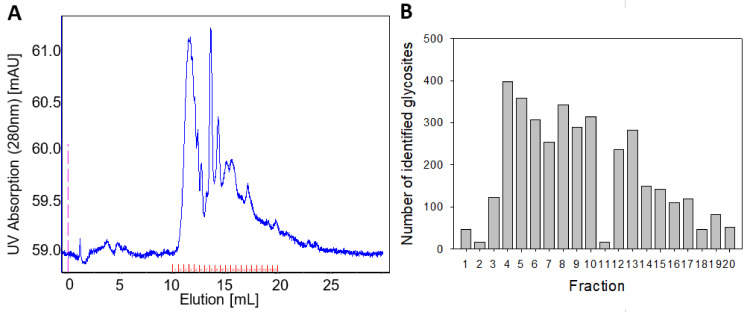
Glycopeptide separation by size exclusion chromatography (SEC) (100–7000 Da). (**A**) Chromatogram includes UV-spectrum at 280 nm (blue) and collected fractions (red). (**B**) Number of identified glycosites with high localization probability (>0.75) in each fraction.

In order to compare N-glycosylation alterations between two samples, glycopeptide fractionation was implemented to separate the same peptides with altered glycosylation into separate fractions. The same peptides but with different glycosylation would be characterized by different mass, hydrophobicity, and charge, and they would appear in different fractions in two different conditions. A comparison of anion exchange, reverse phase, and size exclusion chromatography showed the best glycopeptide fractionation on high resolution size exclusion column (SEC) (Figure 1A, Figure S3A–C, and Supplementary Materials Section 1.3). The resolution power of the SEC peptide separation was analysed using peptide standards in mass range from 3100 to 960 Da and glycopeptides from glycoprotein RNase B. A change glycan length by a single monosaccharide unit would result in a change of ~180 Da for a simple monosaccharide (e.g., glucose, galactose, mannose, and fucose), while sialic acids (e.g., Neu5Ac with a M.W. of ~309 Da) or acetylated derivatives (e.g., N-acetylglucosamine or N-acetylgalactosamine—M.W. of ~221 Da) have higher molecular weights. Therefore, we concluded that the chosen fractionation method allowed for the identification of most glycosylation changes consisting of at least two monosaccharides and, in some cases, even one monosaccharide glycosylation change (details given in Supplementary Materials Sections 1.4 and 2.9).

This study focused on providing information on which proteins and which glycosites show differential glycosylation between two conditions. Since in many cases, a routine mass spectrometry laboratory (such as Core Facility Services) may be focussed on the analysis of unmodified peptides rather than glycopeptides, we aimed to establish a robust strategy that could be easily implemented in collaboration with routine mass spectrometry laboratory. Thus, a deglycosylation step using N-glycosidase F of every fraction, which cleaves all types of asparagine-bound N-glycans and modifies asparagine to aspartic acid as a result, was implemented. The presence of H_2_^18^O ensured that enzyme-induced asparagine deamidation caused a mass shift of 3 Da (instead of 1 Da), which was better identifiable by MS analysis and, in addition would allow us to distinguish glycosites from spontaneous asparagine deamidation that could occur during the previous stages of the experiment. The MS analysis of fractionated and deglycosylated glycopeptides confirmed their good separation between the fractions, as well as the overall capacity of the procedure to identify glycosites (Figure 1B). The optimized procedure for sample preparation thus enabled implementation to compare different cell states. 

### 2.2. Method Implementation

**Figure 2:**
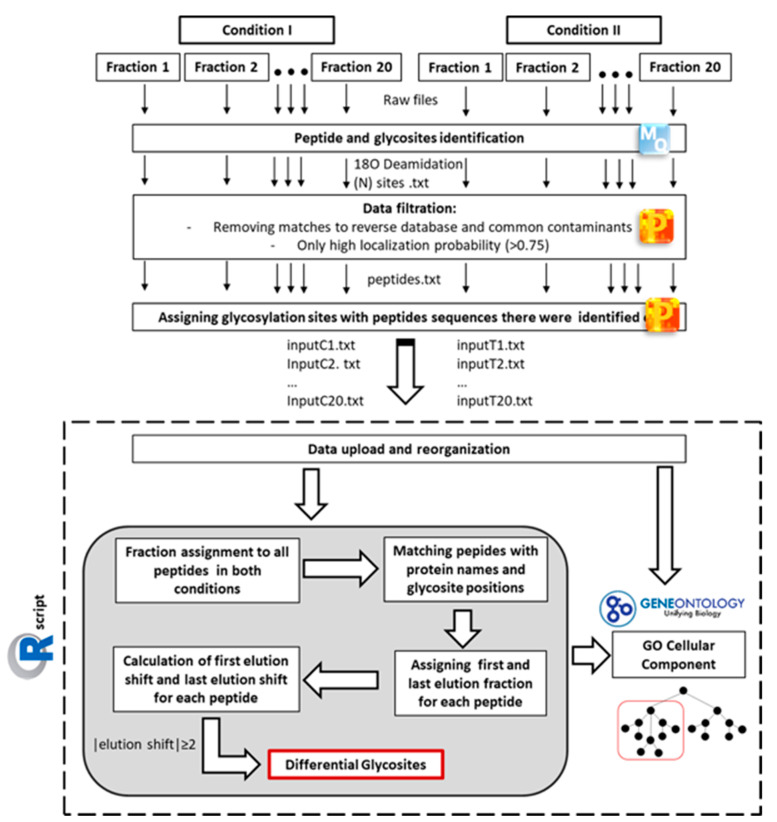
Workflow of mass spectrometric data analysis for differential glycosite profiling. MS raw data from each fraction were separately analysed using the MaxQuant software (MQ). In order to identify glycosites, ^18^O deamidation at asparagine was chosen as variable modification. MQ output was firstly filtered for common contaminants and matches to the reverse database. Only ^18^O deamidation sites with a high localization probability (>0.75) were considered for further analysis. In order to compare glycopeptides sequences, the identified ^18^O deamidation sites were matched with peptide sequence information and the matched data were separately exported as text files for each fraction. For the comparison of two conditions, an in-house R script was used for the data collection, organization, and calculation of elution shifts between the conditions, thus leading to the identification of differential glycosites. Only sites with elution shift ≥ 2 fractions were considered differential. In addition, all glycosites were assigned Gene Ontology cellular component (GOCC) term IDs based on their gene name, which allowed for cellular localization-dependent data analysis.

2-Deoxy-glucose (2DG), is known to modulate protein N-glycosylation in colorectal cancer cells [30]. Therefore, it provided an excellent tool to test the efficacy of our method. Samples from CX1 colorectal cancer cells treated with 4 mM 2DG and untreated cells were prepared as described above. Fractions obtained as described above were subjected to nanoUPLC-coupled mass spectrometry, and the MS raw data underwent analysis, as shown in Figure 2. The first steps of data analysis were performed using the MaxQuant and Perseus software. Firstly, peptides and ^18^O deamidation sites were separately identified in each fraction. The identification data were prefiltered for common contaminants and high localization probability (>0.75) to ensure the highest quality of the data. Subsequently, we aimed to compare the SEC elution shifts of glycopeptides with exactly the same amino acid sequence between both conditions. For that purpose, our versatile in-house R script was used for the further data collection, organization, and calculation of elution shifts between the conditions. The calculated elution shift indicated the shift in molecular weight of the same peptide. Other peptide modifications such as carbamidomethylation, oxidation, and deamidation (NQ) cause minor changes in mass of peptides and thus would not have affected the elution of the glycopeptides in SEC. However, in order to eliminate small technical differences in individual elution profiles from separate chromatographic runs for each condition, only glycosites with elution shift ≥ 2 fractions were considered differential. The elution shift was calculated in both directions, i.e., both earlier and later eluting peptides were marked as differential. As a result, the output from the R script gave comprehensive information on peptide sequence, gene and protein name, glycosite position within the protein, as well as elution fractions, elution shifts, and differential status of the glycosites. In addition, our R script incorporated gene ontology annotations (cellular component; GOCC) from Bioconductor’s org.Hs.en.db annotation package for each gene, which allowed us to analyse and filter data based on the cellular localization of a protein. 

A summary of differential glycosite profiling as obtained from membrane fractions from 2DG vs. untreated cells is shown in Table 1. In total, 1879 ^18^O-deamidation sites (+ 3Da, N-glycosylation sites) with high localization probability were identified on 1695 distinctly identified peptides in all fractions. There were 1331 from identified ^18^O deamidation sites that were known N-glycosylation sites already listed in N-GlycositeAtlas [37] (Table S1). Overall, from the 1066 glycopeptides identified in both conditions, 320 were assigned as differential glycopeptides (marked in Table S1). Peptides identified in only one of the samples (control or 2DG-treated) were not listed as differential. To assess whether differential glycosites are associated with plasma membrane proteins, the sites’ assigned GOCC term IDs were checked for the presence of identifier GO:0005886 for “plasma membrane” or any of its child nodes further downstream in the Gene Ontology cellular component’s tree-like, directed acyclical graph structure. Among differential glycosites, 136 of them came from plasma membrane proteins (Table S2).

**Figure 3:**
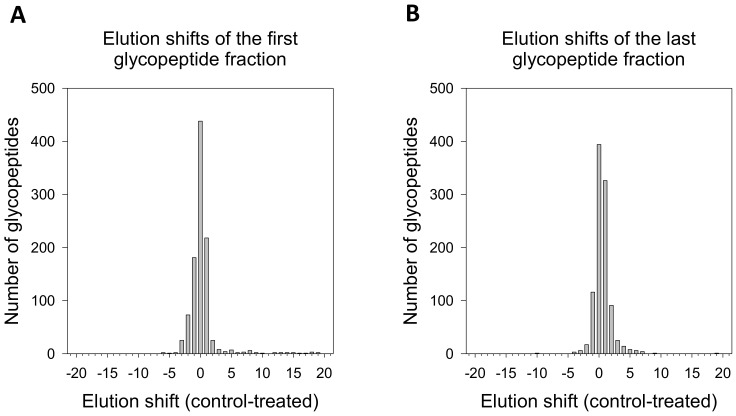
Global analysis of glycopeptide elution shift due to treatment-induced glycosylation. The shift was separately calculated for each glycopeptide. The first (**A**) and last (**B**) glycopeptide fractions are the first and last fractions in which the analysed glycopeptide was identified.

The global analysis of elution shifts (Figure 3) showed that most of the identified glycopeptides showed no significant difference in glycosylation. Elution shifts (≥2 fractions) were observed in both directions. Considering the first elution, 87 peptides were downgraded, i.e., found in later fractions in 2DG-treated cells, and 96 peptides were upgraded, i.e., eluted in earlier fractions in treated cells. In the last elutions, 66 of the peptides were downgraded, while 116 were upgraded. In total, 10% of peptides were found to be upgraded in 2DG-treated cells, while about 7% were downgraded. Furthermore, we observed an almost-twice greater number of glycopeptides in higher elution shifts (≥3) compared to peptides that eluted in earlier fractions. The higher number of peptides eluting in the earlier fraction in 2DG-treated cells compared to control indicated the higher molecular weight of 2DG-induced N-glycosylation structures.

### 2.3. Overview of the Differential Glycosite Profiling Strategy

**Figure 4:**
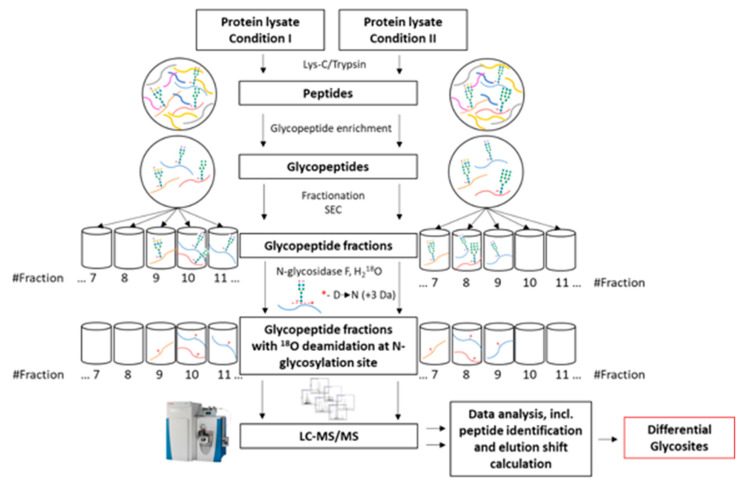
Differential glycosite profiling strategy. Membrane protein lysates underwent enzymatic digestion using a combination of Lys-C and trypsin followed by glycopeptide enrichment. Samples enriched in glycopeptides were then fractionated on SEC into 20 fractions. Each fraction was deglycosylated using N-glycosidase F in ^18^O-water to ensure a +3 Da shift during the deglycosylation-induced deamidation of asparagine. Furthermore, each fraction was analysed by mass spectrometry (LC–MS/MS). Through complex data analysis, which led to a comparison of the elution times of the same glycopeptides in both conditions, differential glycosites were identified.

A versatile mass spectrometry-based strategy was developed to compare protein N-glycosylation alterations (Figure 4). The proposed procedure consists of 6 main steps:

1. Membrane protein extraction and enzymatic digestion
2. Glycopeptide enrichment
3. Glycopeptide fractionation
4. Glycopeptide deglycosylation
5. Mass spectrometric analysis (LC–MS/MS)
6. Data analysis

Each of them were optimized in order to ensure efficiency and high-quality data. The procedure was successfully applied to a representative model system.

## 3. Discussion

Considering the estimated more-than 7000 glycans linking tens of thousands of glycosites on proteins in humans, it appears an enormous challenge to profile whole human glycoproteomes [38]. Moreover, glycans are the only biomolecules that can build many different oligomers from few building blocks [39]. A calculation of all possible oligosaccharide isomers yields 1.05 × 10^12^ structures for a reducing hexasaccharide [40]. Thus, the complete glycome analysis of a human cell appears to be a nearly intractable puzzle. Nevertheless, various strategies applying mass spectrometry in combination with prefractionation techniques have been developed to approach this demanding task, and these are now considered core technologies in glycoproteomics research [41,42]. Various prefractionation techniques that target glycan structures, including solid phase extraction based on hydrazide capturing, lectin affinity, separation techniques applying porous graphite carbon or boronic acid, and hydrophilic interaction, have been established [41]. However, even after applying these protein glycosylation-targeting enrichment technologies, a very complex sample composition remains to be analysed. Accordingly, even after efficient prefractionation, screening glycoproteomes to find changes that are related to changes in a cell’s behaviour is particularly challenging. 

Our experimental strategy aims to provide a versatile tool to focus on glycoproteins where significant changes of glycosylation occur in response to physiological or pathological events. As highlighted by cancer-associated glycoproteome alterations, such glycan changes may take a variety of forms: loss of expression or excessive expression of certain glycans, increased expression of incomplete or truncated glycans, and altered terminal sialylation and fucosylation. On the other hand, the appearance of completely novel glycans is a rather rare event [34]. The most common structural changes may result in altered the chromatographic behaviour of affected glycopeptides in ion-exchange-, size-exclusion-, or reversed-phase-chromatography. Therefore, analysing the chromatographic behaviour of glycopeptides using a high-resolution chromatography system would help to detect affected glycopeptides. Comparing the mentioned chromatographic methods indicated SEC as the most effective fractionation tool of a glycopeptide pool after the digestion of a membrane protein extract and lectin prefractionation. 

In order to test the optimized fractionation strategy in combination with the mass spectrometric identification of altered glycopeptides, the SEC elution profiles after the prefractionation of two differential glycoproteomes obtained from untreated CRC cells and 2DG-treated cells were compared. An analysis of the fractions by mass spectrometry in tandem with our R-script proved the effectiveness of the method to identify differential glycopeptides. 

Glycopeptides that appeared in only one condition were not listed as differentially glycosylated, since it was not possible to decide whether their absence in the other condition was due to the loss of ConA target structures in their glycan part or the complete loss of glycosylation. It could also have been caused by the missing expression of the respective protein. Moreover, the complexity of proteomic instrumentation for LC–MS/MS introduces many possible sources of variability. In particular, single peptide detection could be variable due to technical limitations [43]. In order to ensure that we only listed glycosites with proven glycan alterations, we had to accept that we might have neglected some glycopeptides where it was not possible to distinguish between biological or technical glycopeptide loss. 

Though our method implementation experiment focused on changes in N-glycosylation in membrane proteins, the method could be easily adapted to other types of glycosylation or specific glycan structure motifs. Lectin-based solid phase extraction targeting O-glycopeptides could be, for example, be achieved with Jacalin or peanut agglutinin. Likewise, more specific extraction, e.g., focusing on sialylated or fucosylated peptides, could by accomplished by using the *Sambucus nigra*, *Maackia amurensis*, and *Aleuria aurantia* lectins [44]. As an alternative, specific structures like glycan tumour markers can be selected for by glycan-specific antibodies [45].

Our method certainly does not lay claim to completely cover all changes in a cellular glycoproteome; rather, it presents a “rough filter” to detect glycosites where substantial changes of at least two monosaccharides occurred. However, in the current state of glycobiological research, where the enormous complexity of possible changes is still overwhelming, initial focus on glycoproteins and glycosites with major changes might be favourable. Thus, we consider the screening method for markedly altered glycosites an ideal starting point for more detailed investigations on the functionality and detailed structure of the affected glycoprotein. It should be kept in mind that proposed methodology, like any screening strategy, requires further complementary validation. 

In general, we envision diverse application possibilities for our method in glycobiology. Different starting materials, including whole protein lysates, tissue extracts, subcellular fractions, and body fluids, can be processed. Our bioinformatical tool to analyse such complex data proved to be an adaptable and versatile solution to compare glycoproteomes in any given number of fractions between two conditions. If subcellular fractions are of particular interest, bioinformatic annotation may be included in data processing, e.g., for the specific targeting of plasma membrane-associated glycoproteins as shown in the presented method implementation. The annotation of glycosites with the respective genes’ Gene Ontology cellular component (GOCC) term identifiers allows for the mapping of them to the tree-like, acyclical graph of ontology and provides an elegant solution for filtering the result set by comparing associated GOCC IDs to whole “branches” of the ontology that consist of named nodes and all of their subsequent children [46,47]. This approach makes it possible to vary the scope of interest from a very broad focus on a global cellular level down to very specific cellular compartments by specifying root nodes for the branches at the desired position and height of the GOCC tree. Compared to, e.g., simple string matching for words of interest in text-based annotations, this approach is both more robust and comprehensive because it makes use of the curated nature of the annotations and the defined relational structure of the ontology. The strategy could be combined with different labelling strategies, such as dimethyl labelling [48] and TMT [49], to provide quantitative analysis and/or to compare more than two conditions. Our methodology could be implemented together with modern gene technologies, like genetic transformation, gene knockout, and epigenetic manipulation, thus enabling screening for the effects of gene activation on a cell’s glycoproteome. Additionally, the influence of disease-associated mutations, e.g., mutations in tumour-driver or tumour-suppressor genes in cancer cells or tissues, may be explored. 

N-glycosylation is a multistep process not only consisting of the simple assembly of monosaccharide units but also involving complex glycan trimming to pave the way for final maturation of the protein-bound glycan structure [2]. Thus, the interference of 2DG with a whole biosynthetic sequence will not only cause the truncation of the N-glycans but also potentially give rise to new structures, thereby also resulting in glycopeptides carrying elongated mature glycans [30,50], as observed in our study. Detecting such major disease- and treatment-associated glycome changes is considered a key to new diagnostic markers and therapeutic targets [14]. For example, immunotherapy is considered one of the most promising therapy options in cancer patients [51]. However, the broad application of such therapy strategies requires well-defined and highly potent target antigens. Glycan-based cancer neoantigens are increasingly considered to be novel targets in the immunotherapy of tumours [52]. Finally, small molecules that interfere with glycosylation or act as glycan-mimetics are currently tested for their potential as drugs to treat common diseases, like cancer, infection and inflammation [22,53].

Altogether, glycobiological and glycopathological research requires a broad repertoire of different methods that work hand-in-hand to drive this highly topical research field forward. Based on the successful testing of our strategy with a cell culture model, we suggest our method to screen biological samples for proteins carrying altered glycosides as a useful module in various experimental settings that target glycan functions in health and disease.

## 4. Materials and Methods

### 4.1. Cell Culture and Membrane Extraction

The colorectal cancer cell line (CX1) was obtained from the German Cancer Research Center Repository and cultured in Dulbecco’s Modified Eagle’s Medium (DMEM) supplemented with 10% foetal bovine serum (FBS), 1 mM L-glutamine, 100 U/mL penicillin, 100 μg/mL streptomycin (Life Technologies, Karlsruhe, Germany) at 37 °C in a 5% CO_2_ atmosphere. After 72 h of culture in the presence (treated) or absence (control) of 4 mM 2DG, the cells were harvested by scrapping in cold PBS. After centrifugation, the cells were suspended in a 20 mM Tris buffer at pH 7.4 with protease inhibitors (cOmplete Mini; Roche, Basel, Switzerland), followed by sonication (30 s) and centrifugation (15,000× *g*, 4 °C, 7 min). The insoluble fraction (membrane fraction) was resuspended in a RIPA buffer. After overnight incubation on the rotator at 4 °C, the sample was centrifuged (15,000× *g*, 4 °C, and 7 min). The soluble fraction was collected, and the protein concentration was determined with a Bradford assay (Bio-Rad, Vienna, Austria) according to the manufacturers’ instructions.

### 4.2. Membrane Protein In-Solution Lys-C/Tryptic Digestion in NH_4_HCO_3_/Urea 

First, 0.5 mg of extracted membrane fraction of treated and control cells was precipitated by quantitative protein precipitation using a methanol–chloroform–water mixture [54] in order to remove reagents prior to tryptic digestion. Precipitated protein samples were redissolved in a 150 µL digestion buffer (6 M urea, 100 mM NH_4_HCO_3_) by 1 h incubation at 25 °C for at least 1 h. Samples were first treated with 5 µL of 1 M dithiothreitol (DTT) in a digestion buffer at 45 °C for 1 h to completely reduce disulphide bonds. The resulting thiol groups were then alkylated by adding 7 µL of 0.5 M iodoacetamide (IAA) in a digestion buffer followed by 30 min of incubation in the dark at 25 °C. After adding 5 μL of a 1 M DTT-containing solution, the mixture was incubated for 15 min at 37 °C to let IAA react with a thiol group. The digestion of 0.5 mg of membrane protein was performed firstly with 3.75 μg Lys-C (Promega, Walldorf, Germany) in 100 mM NH_4_HCO_3_. After overnight incubation at 37 °C with constant gentle shaking, the samples were diluted with 700 µL of 100 mM NH_4_HCO_3_ and 0.125 mM CaCl_2_ to achieve the UREA concentration (~1M) required for the perseverance of tryptic activity. The samples were further digested overnight at 37 °C by 10 μg of trypsin (Promega) in 100 mM NH_4_HCO_3_ and 0.125 mM CaCl_2_. In total, 4 mg of membrane protein (8 × 0.5 mg) were digested for each condition (treated or control) for glycosite profiling. 

### 4.3. Stage Tipping

Samples were stage tipped as described in [55,56]. Briefly, for each sample, 4 mg of a reverse phase material (Oligo^TM^ R3; Applied Biosystems, Foster City, CA, USA) slurry in ddH_2_O:acetonitrile (1:1, *v*:*v*) were retained in the pipette tip by a small portion of C18 material (Agilent Technologies, Santa Clara, CA, USA) conditioned by acetonitrile. Material was equilibrated with 2.5% formic acid. Samples were acidified by the addition of 10% formic acid to a final concentration of 2% and slowly passed through the material (with the addition of 2.5% formic acid to reach working volume). After five washing steps with 2.5% formic acid, elution was performed twice with 0.6% acetic acid in 80% acetonitrile. The working volumes of all solutions were adjusted to 100 μL. 2 mg (4 × 0.5 mg) of tryptic digests were pulled together and dried completely in a vacuum centrifuge for glycopeptide enrichment.

### 4.4. Glycopeptide Enrichment

Glycopeptide enrichment using streptavidin-bound magnetic beads coupled with biotinylated ConA was performed based on previously described protocol [57] and adjusted to peptide samples. In order to extract glycopeptides from 2 mg of tryptic digest, 0.5 mL of Dynabeads MyOne Streptavidin T1 (Thermo Fisher Scientific, Karlsruhe, Germany) were washed 5 times with a 1 mL Tris buffer (20 mM Tris (pH 7.4) and 500 mM NaCl) and incubated with 250 µg of biotinylated ConA (50 µL; Vector Laboratories, Burlingame, CA, USA) in a 250 µL Tris buffer. After 2 h incubation on a rotator at RT, the beads were washed in a 1 mL Tris buffer and a 1 mL Tris buffer with 1% Triton, followed by 3 washings with a 1 mL Tris buffer. Following tryptic digestion, 2 mg samples were redissolved in a 1 mL binding buffer (20 mM Tris (pH 7.4), 150 mM NaCl, 1 mM MnCl_2_, and 1 mM CaCl_2_) by short sonication and 30 min incubation at RT with gentle shaking and added to prepared ConA beads. The samples were incubated overnight on a rotator at 4 °C. The next day, the beads were washed 5 times with a 1 mL binding buffer, and membrane glycopeptides were eluted 3 times with a 0.5 mL binding buffer and 0.5 M methyl-mannopyranoside and incubation for 1 h on a rotator at 4 °C. For each condition, 2 × 2 mg underwent glycopeptide enrichment. The samples were stage tipped as described above (Section 4.3), and samples from each condition were pulled together (treated and control separately).

### 4.5. Glycopeptide Fractionation

Glycopeptides from each condition (treated and control) were resuspended in a 50 µL solution of 70% PBS (phosphate-buffered saline) and 30% acetonitrile by sonication and 30 min incubation at RT with gentle shaking. Glycopeptides were further fractionated on a Superdex^TM^ 30 Increase 10/300 GL size exclusion column (Merck, Darmstadt, Germany) using an ÄKTA purifier (GE Healthcare, Chicago, IL, USA). Separation was performed in a solution of 70% PBS and 30% acetonitrile for 30 mL at a flow rate of 0.1 mL/min. In each run, 20 fractions (0.5 mL each) were collected based on UV-spectrum at A280 nm after 10 mL and up to 20 mL elution. All collected fractions were completely dried in a vacuum centrifuge.

### 4.6. Glycopeptide Deglycosylation

The samples were resuspended in 15 µL of 50 mM NH_4_HCO_3_ in H_2_^18^O by incubation for 30 min at RT with gentle shaking. To each sample, 1 µL of N-glycosidase F (Roche) (2U/µL in 50 mM NH_4_HCO_3_ in H_2_^18^O, Roche) was added and followed by overnight incubation at 37 °C. All the samples were stage tipped as described above (Section 4.3) prior to LC–MS/MS analysis.

### 4.7. LC–MS/MS

Dried peptides were reconstituted prior to LC–MS/MS analysis in 2.5% 1,1,1,3,3,3-Hexafluoro-2-propanol, 0.1% TFA in water or 50 mM citrate, and 0.1% TFA. First, the peptides were loaded on a trapping cartridge (Acclaim PepMap300 C18, 5 µm, 300 Å-wide pore, Thermo Fisher Scientific) and desalted for 3 min using 0.05% TFA. Peptide separation was performed using a multistep gradient of buffer A (0.1% formic acid in water) and buffer B (0.1% formic acid in acetonitrile), with the main step ramping buffer B concentration from 5% to 30% over 43 min on a nanoEase MZ peptide analytical column (300 Å, 1.7 µm, 75 µm × 200 mm, Waters) using an UltiMate 3000 UHPLC system (60 min of total analysis time). Eluting peptides were subsequently analysed by an Orbitrap Exploris 480 mass spectrometer (Thermo Fisher Scientific, Karslruhe, Germany) running in its data-dependent acquisition mode where one full scan (380–1400 *m*/*z*, 3e6 ions AGC target, maxIT of 45 ms) at a 60k resolution was followed by MS/MS scans for a 1 sec cycle time. Precursors were isolated with 1.4 *m*/*z*, peptides were fragmented using 26 NCE, and MS/MS scans were recorded at a 15k resolution (1e5 ions AGC target, maxIT of 54 ms). Unassigned and signals were excluded from fragmentation, and dynamic exclusion was set to 35 sec for 2–6x charged features.

### 4.8. Peptide and Glycosite Identification

The MS files were separately processed with the MaxQuant software (1.6.14) [58] and searched with the Andromeda search engine [59] against the human SwissProt database (download: 15 June 2020; 20,365 entries). Enzyme specificity was set to that of trypsin, allowing for the N-terminal cleavage of proline residues and up to four missed cleavage sites (for proteome analysis). A minimum peptide length of seven amino acids was required. Carbamidomethylation (C) was set as a fixed modification, whereas oxidation (M), deamidation (NQ), protein N-terminal acetylation, and deamidation 18O (N) were considered variable modifications. Mass tolerances were defined for precursor and fragmented ions as follows: MS first search—20 ppm; MS main search—6 ppm; MS/MS match tolerance—20 ppm; and MS/MS deisotoping tolerance—7 ppm. The false discovery rates at the protein and peptide levels were set to 1%.

### 4.9. Data Analysis

Data analysis was performed with the Perseus (version 1.6.14) software [60]. For each fraction, a ‘deamidation 18O (N)Sites.txt’ output table was uploaded to the Perseus (version 1.6.14) software while adding ‘peptide IDs’ column as a text column. Matches to the reverse database and common contaminants were removed from the MaxQuant output. Each 18O deamidation site with a localization probability higher than 0.75 was matched with a peptide sequence from ‘peptide.txt’ using ‘peptide IDs’. Tables containing at least protein names, gene names, and peptide sequences were exported as text files with corresponding condition identifiers and fraction numbers, e.g., T13—fraction 13 of the treated sample. Identified 18O (N) deamidation sites were compared with the human N-glycosite database of N-GlycositeAtlas (downloaded on 10 February 2021) [37]. 

The further processing of the data exported from Perseus was done in the R statistical programming language [61] using the RStudio IDE [62]. In brief, all exported text files were read in a combined data frame using the readr package [63]. The sample group (control or treatment condition) was extracted from the non-numeric part at the beginning of the file name (i.e., “C” or “T”), and the numeric part of the file name was interpreted as the fraction number. File name parsing was implemented using the stringr package [64]. The general handling of the imported data was using packages from the tidyverse set of packages [65], specifically the packages tidyr [66], dplyr [67], purrr [68], and tibble [69].

Entries in the data frame that contained multiple peptide sequences per row were split into a single row per sequence, copying all further columns’ information for the newly created entries from the original row. 

A copy of the data was then split by sample group; the peptide sequence and sample fraction were selected and reformatted into lists in “wide format,” resulting in a matrix-like structure with the sample fractions in one dimension and the corresponding peptide sequences in the other dimension.

A vector containing all unique peptide sequences that were present in this matrix was generated, and each entry in this vector was matched back against the matrix to obtain the fractions it appeared in. The result was a key-value list for each of the sample groups with each unique peptide sequence as key and a vector containing the fraction numbers where it was detected in as value.

For the final table of results, a “tibble” containing the columns with the gene names, protein names, position, and all unique peptide sequences from the initial data frame was initialized. For both treatment conditions, the vectors containing the sample fractions for each unique peptide sequence were extracted from the key-value lists created earlier and appended as two columns. Minimum and maximum fraction numbers were calculated per condition and for all sequences, and the difference between control and treatment sample groups’ minimum and maximum fraction number was obtained.

Based on the absolute difference for either the minimum or maximum fraction number larger than 1 between sample groups, peptides were classified as differentially glycosylated and the result of the classification appended to the tibble, encoding Boolean values of “True” and “False.” Wherever a peptide sequence was completely absent in one of the sample groups, the corresponding entry and all derived data (minimum and maximum fractions, differences and glycosylation status) were coded as NA.

GOCC term IDs and associated gene symbol names were extracted from the R Bioconductor human organism annotation package org.HS.eg.db, version 3.11.4 [70] and matched to the gene names provided by the Perseus software for each peptide. Whenever there were multiple gene names present for a peptide, the first non-empty GOCC ID result set returned was used. Additionally, the IDs were mapped to their textual representation for inclusion in result output tables.

The R Bioconductor GO.db annotation package version 3.11.4 [71] was used and queried to obtain the set of GOCC ID “GO:0005886” for the cellular component term “plasma membrane” and all of its “offspring” (direct as well as indirect children) nodes on this branch of the ontology. 

Each of the GOCC IDs that were associated with a peptide were checked for whether they were present on the GOCC “plasma membrane” ontology branch, thereby providing the means for filtering the peptide result set for plasma membrane-associated entries.

A tibble was exported to a text file containing data for all unique peptides (Table S1), and a tibble that was filtered for differential peptides associated with the cellular plasma membrane (Table S2) was exported to the comma-separated-value-format (CSV). 

All glycan structures were drawn using the DrawGlycan-SNFG online tool [72]. 

## Figures and Tables

**Table 1:** Summary of differential glycosite profiling of 2DG vs. untreated control cells.

	# Total
Identified ^18^O deamidated sites	1879
Known glycosites ^1^	1331
Identified individual glycopeptides in all fractions	1820
Glycopeptides in both conditions	1066
Differential glycopeptides	320
Differential plasma membrane glycopeptides ^2^	136

^1^ Compared with N-Glycosite Atlas; ^2^ Cellular localization based on Gene Ontology Cellular Component (GOCC) annotation.

## Data Availability

The mass spectrometry proteomics data have been deposited to the ProteomeXchange Consortium (http://proteomecentral.proteomexchange.org accessed on 16 March 2021) via the PRIDE partner repository [73] with the project accession: PXD024770. Reviewer account details: Username: reviewer_pxd024770@ebi.ac.uk Password: 1sKLZqDn.

## Supplementary Materials

The following are available online: Supplementary Information: Figure S1: Efficiency assessment of glycopeptide enrichment strategies using ConA. Figure S2: Comparison of protein digestion strategies for membrane proteome. Figure S3: Comparison of different glycopeptide separation strategies. Table S1: Differential glycosite profiling of 2DG-treated colorectal cancer cells in comparison with untreated control. Table S2: Result set from Table S1 filtered to include only differential peptides associated with the GOCC GO:0005886 “plasma membrane” ontology branch. Table S3: Size exclusion chromatography (SEC) assessment using Rnase B glycopeptides. Table S4: Size exclusion chromatography (SEC) assessment using Peptide Standard. R script: Differential glycosite profiling.
