# Supplementary material for: Differential Glycosite Profiling—A Versatile Method to Compare Membrane Glycoproteomes
Source: Molecules. 2021 Jun 10;26(12):3564. doi: 10.3390/molecules26123564 (PMC8230608; doi:10.3390/molecules26123564)
Supplement: Supplementary file 1 [file molecules-26-03564-s001.zip › Supplementary Information.pdf]

1. Supplementary Results

1.1 Comparison of membrane protein enzymatic digestion strategies

Four different strategies for membrane protein enzymatic digestion were compared:

1. in-solution tryptic digestion in NH_4HCO_3 – buffer
2. filter-aided sample preparation (FASP) [1]
3. in-solution Lys-C/tryptic digestion on S-Trap™
4. in-solution Lys-C/tryptic digestion in the presence of urea
5. in-solution Lys-C/tryptic digestion in NH_4HCO_3/Na-deoxycholate

While method nr 1. proved to be ineffective most likely due to low protein solubility, FASP applied to higher amounts of poorly soluble membrane proteins also lead to its precipitation on the filter (Figure S1A). Comparing methods 3.-5. indicated that use of Na-deoxycholate and its removal in the end of enzymatic digestion caused simultaneous peptide and protein precipitation as shown on Figure S1B (lanes: 3 and 4). Methods 3. And 4. revealed similar digestion efficiency (FigureS1B-D). Although, an important advantage of S-Trap™ is time efficiency, the whole procedure requires higher amounts of enzymes (Lys-C: 2x more and Trypsin: 5x more) and thus is less cost effective. Furthermore, elution from S-Trap™ requires presence of organic phase (at least 50% acetonitrile) which seems to not be well compatible with our glycopeptide enrichment strategy. Even after vacuum centrifuging the sample showed very low pH and required purification by Stage Tipping before glycopeptide enrichment. This resulted in a time of sample preparation close to Lys-C/Trypsin in-solution digestion in the presence of urea (data not shown). All things taken together, in-solution Lys-C/Trypsin digestion in presence of urea, followed by Stage Tipping preparation for the subsequent glycopeptide enrichment became method of choice in our differential glycosite profiling strategy.

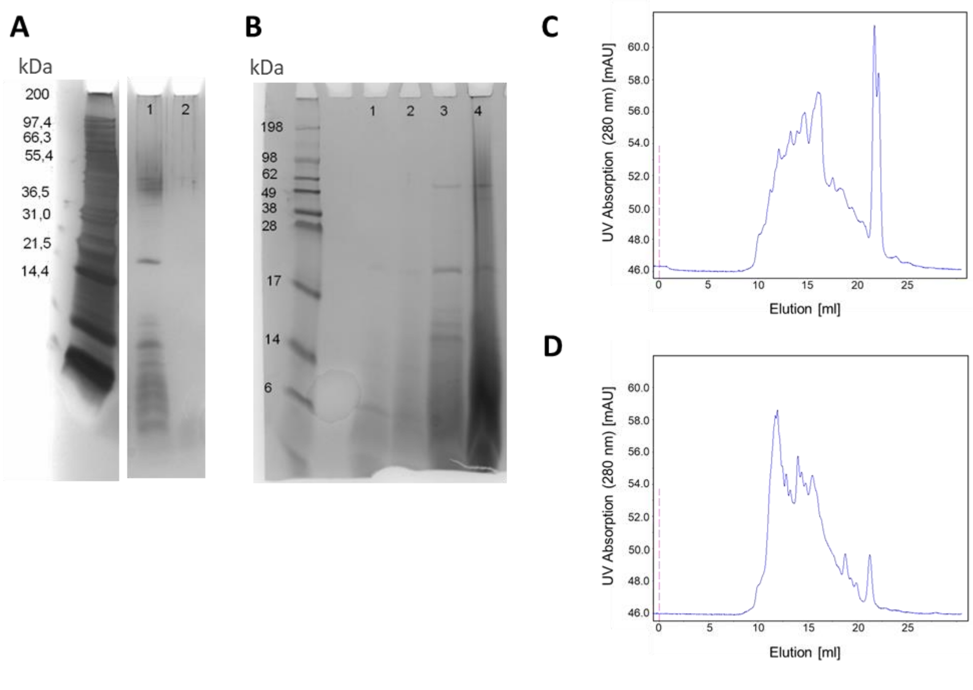

Figure S1. Comparison of protein digestion strategies for membrane proteome. All strategies are described in Methods (section 4.2) and Supplementary Methods (sections 2.1-2.4). **A**. Silver stain of two membrane digestion startegies: 1. - In-solution tryptic digestion in NH_4HCO_3; 2. – FASP tryptic digestion. **B.** Coomassie stain of 3 membrane digestion startegies: 1. - In-solution Lys-C/tryptic digestion on STrap; 2. – In-solution Lys-C/tryptic digestion in NH_4HCO_3/Urea. 3. and 4. - In-solution Lys-C/tryptic digestion in NH_4HCO_3/Na-deoxycholate. After Na-deoxycholate precipitation supernatant (3) and redissolved pellet (4) were loaded on the gel. **C.** and **D**. Separation in Size Exclusion Chromatography (100-7000 Da). **C.** 2 mg of Lys-C/Tryptic digestion using STrap. **D.** 2 mg of in-solution Lys-C/Tryptic digestion. Chromatograms include UV-spectrum at 280 nm (blue).

1.2 Comparison of glycopeptide enrichment methods

Two common solid phase extraction methods were compared: ConA-bound sepharose suspension (Merck, Darmstadt, Germany) or streptavidin-bound magnetic beads coupled with biotinylated ConA [2].

The first strategy, although more cost effective and faster, showed high levels of ConA leakage and thus presented strong background which would impact further steps and quality of the sample (Figure S2). Thus, streptavidin-bound magnetic beads coupled with biotinylated ConA proved to be the better strategy for enrichment of N-glycosylated peptide

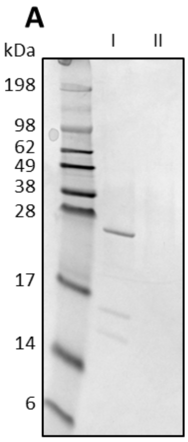

B

Fraction	214 nm	
	Peptide sample	Beads control
Binding Buffer	0.212	
Flow Through	2.839	1.314
Last Washing Step	0.335	0.269
Elution Buffer	0.352	
1. Elution	0.895	0.838
2. Elution	0.824	0.776
3. Elution	0.860	0.801

C

Fraction	214 nm	
	Peptide sample	Beads control
Binding Buffer	0.209	
Flow Through	3.098	0.384
Last Washing Step	0.200	0.216
Elution Buffer	0.384	
1. Elution	0.673	0.342
2. Elution	0.410	0.353
3. Elution	0.386	0.366

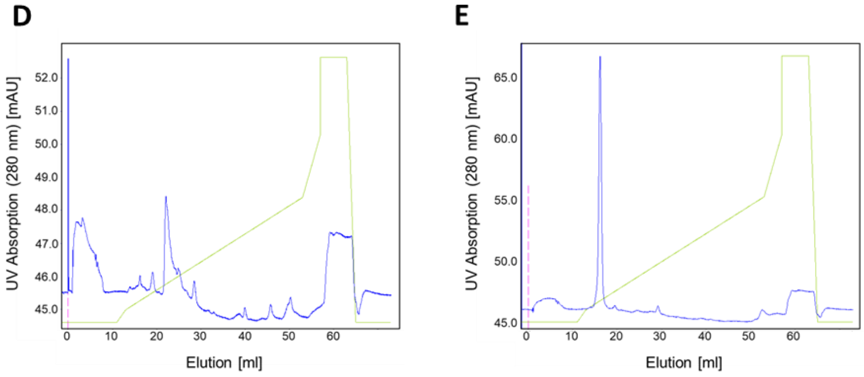

Figure S2. Efficiency assessment of glycopeptide enrichment. Compared strategies used 2 mg of peptide samples after tryptic digestion as starting material and are described in Methods (section 4.4) and Supplementary Methods (section 2.5). **A.** SDS-PAGE and Coomassie staining of glycopeptide enrichment elutions coming from ConA-bound agarose beads (I) and streptavidine-bound magnetic beads and biotinylated ConA (II). Visible band in lane I is probably ConA leakage (Monomer MW: 25.5 kDa). **B.** and **C**. Tables summarizing the glycopeptide enrichment with agarose (**B**) and magnetic (**C**) beads. At wavelength of 214 nm the peptide bond has a maximum of absorbance. High values visible in agarose beads control confirm the ConA leakage as well as influences the glycopeptide enrichment read out, which makes it impossible to assess its efficiency. **D.** and **E.** Separation of pooled elutions from the glycopeptide enrichment with agarose (**D**) and magnetic (**E)** beads using reverse phase column with step wise gradient of (green) described in Supplementary Methods. UV absorption measurement at 280 nm (blue line) shows strong background from agarose beads in the elution compared to magnetic beads.

1.3 Comparison of glycopeptide fractionation

Comparison of anionexchange, reverse phase and size exclusion chromatography showed the best glycopeptide fractionation on high resolution size exclusion column (SEC) (Figure S3A-C).

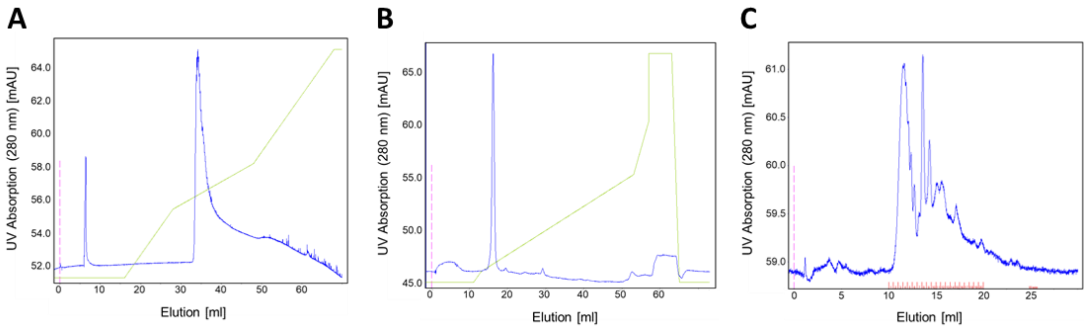

Figure S3. Comparison of different glycopeptide separation strategies. All strategies are described in Methods (section 4.5) and Supplementary Methods (sections 2.6 and 2.7). A. Glycopeptide separation using Anion Exchange column. Chromatogram includes UV-spektrum at 280 nm (blue), gradient of 0 – 1M NaCl in 20 mM Tris, pH 8.0 (green). B. Glycopeptide separation using Reverse Phase column. Chromatogram including UV-spectrum at 280 nm (blue) and gradient of acetonitrile concentration from 2% to 85% (green). C. Glycopeptide separation by Size Exclusion Chromatography (SEC) (100 – 7000 Da). Chromatogram includes UV-spectrum at 280 nm (blue) and collected fractions (red).

1.4 Peptide Standard and RNase B glycopeptide analysis

In order to analyse the resolution power of chosen fractionation strategy (Size Exclusion Chromatography) elutions of as 3 glycopeptides coming from digestion of RNase B (Table S3) as well as 18 defined peptide standards in mass range from 3100-960 Da (Table S4) were analysed. No difference between the replicates of peptide standard has been observed while using the cut off of ≥2 elution shift between the peptides. Slight differences in elution between the two technical replicates were noted as well as smearing of abundant peptides into the more fractions. However, there were visible differences in elutions between the peptides of different molecular weight, especially in the fractions of the first elution. Analysing RNase B glycopeptides allowed on distinguishing the two analysed glycopeptides and led to similar observations. Therefore, the described method allows the identification of most glycosylation changes consisting of at least 2 monosaccharides. Observed resolution could also lead to distinguishing between single monosaccharide glycosylation changes in some cases.

2. Supplementary Methods

2.1 In-solution tryptic digestion in NH_4HCO_3 – buffer

In solution tryptic digestion in NH_4HCO_3 – buffer was performed as described before [3]. Precipitated protein samples (500 µg) were redissolved in 135 µl 40 mM NH_4HCO_3 solution by pipetting and at least 1 h incubation at 25 °C in a thermomixer (600 rpm). Samples were first treated with 1 µl of 0.37M dithiothreitol (DTT)-containing solution in 40 mM NH_4HCO_3 at 45 °C for 1 h to completely reduce disulfide bonds, the resulting thiol groups were then alkylated after adding iodoacetamide (IAA)-containing solution in 40 mM NH_4HCO_3 (2 µl 0.37 M IAA) and 30 min incubation in the dark at 25 °C. After adding 1 µl of respective DTT-containing solution, the mixture was incubated for 15 min at 37 °C to let IAA react with a thiol group. Digestion was performed with 5 µg trypsin in 40 mM NH_4HCO_3 solution overnight at 37 °C with constant shaking on a Thermomixer (500 rpm). Next day, another 5 µg of trypsin in 40 mM NH_4HCO_3 solution was added to 0.5 mg protein sample and incubated for at least 3 h at 37 °C with gentle shaking. 5 µg from 0.5 mg sample was analyzed by SDS-PAGE.

2.2 FASP tryptic digestion

Tryptic digestion using filter-aided sample preparation (FASP) was done based on Wisniewski et al. [1]. Precipitated protein sample (1 mg) was redissolved in 250 µl 100 mM NH_4HCO_3 containing 6M Urea by pipetting and at least 1 h incubation at 25 °C in a thermomixer (600 rpm). The sample was then treated with 2.5 µl 1M dithiothreitol (DTT)-containing solution in ddH_2O and incubated at 37 °C for 1 h to completely reduce disulfide bonds, the resulting thiol groups were then alkylated after adding 7 µl of 0.5 M iodoacetamide (IAA)-containing solution and 30 min incubation in the dark at 25 °C. After adding 2.5 µl of

1M DTT-containing solution, the mixture was incubated for 15 min at 37 °C to let IAA react with a thiol group. The sample was further transferred onto centrifugal filter (YM-30, Burlington, MA, USA) and centrifuged at 13000 rpm for 15 min. Further the sample was washed three times with 100 µl of 20 mM Tris pH 8.5 and digested with 10 µg trypsin in 60 µl volume at 37 °C during an overnight incubation at wet chamber. Theoretical 5 µg from digestion mixture was analyzed by SDS-PAGE.

2.3 *In-solution Lys-C/tryptic digestion on S-Trap™*

Membrane protein digestion using S-Trap™ midi (ProtiFi, Huntington, NY, USA) was performed according to manufacturer's instructions. Briefly, precipitated protein samples (1 mg) were redissolved in 5% SDS, 50 mM TEAB pH 7.55 by 30 min. incubation at RT with gentle shaking. The sample was further reduced and alkylated as in section 2.2. and centrifuged to remove undissolved matter. Further, 12% phosphoric acid was added to obtain final concentration of 1.2%. In the next step, 3.3 ml 90% methanol, 100 mM TEAB, pH 7.1 was added and formation of colloidal protein particles was observed. The sample was mix thoroughly and transferred onto the S-Trap™ midi and centrifuged at 4000 g for about 1 min. The flow through was removed and the S-Trap was washed 6 times with 90% methanol, 100 mM TEAB, pH 7.1. After the transfer to a new tube, 100 µg trypsin and 15 µg Lys-C in 500 µl 50mM TEAB, pH 7.4 in total were added onto the S-Trap. Solution was centrifuged into the membrane (short spin). The flow through was added again onto the column. The sample was placed in the preheated water bath at 37 °C and incubated overnight with cap placed gently on the falcon (in order to equalize the pressure). Next day the peptides were eluted by centrifugation using three different solutions: 500 µl 50mM TEAB, pH 7.4, 500 µl 0.2% formic acid and 500 µl 50% Acetonitrile, 0.2% formic acid. For next steps, sample was dried completely in a vacuum centrifuge. About 10 µg were analyzed by SDS-PAGE and tryptic digestion of 2 mg membrane protein was analyzed by Size Exclusion Chromatography.

2.4 *In-solution Lys-C/tryptic digestion in NH_4HCO_3/Na-deoxycholate*

Precipitated protein sample (1 mg) was redissolved in 150 µl 100 mM NH_4HCO_3-buffer containing 1% Na-deoxycholate and 0.125 mM $CaCl_2$ by pipetting and at least 1 h incubation at 25 °C in a thermomixer (600 rpm). Samples were reduced and alkylated as described in section 2.2. Digestion was performed with 20 µg trypsin in 100 mM NH_4HCO_3-buffer containing 0.125 mM $CaCl_2$ overnight at 37 °C with constant shaking on a Thermomixer (500 rpm). Next day, 6 µl of 10% formic acid was added to the sample in order to precipitate Na-deoxycholate. Further, the sample was centrifuged (short spin) and supernatant was collected and dried in a vacuum centrifuge. Both whole supernatant and pellet were further analyzed by SDS-PAGE.

2.5 *Glycopeptide enrichments using ConA-bound agarose beads*

In order to extract glycopeptides from 2 mg of tryptic digest 0.5 ml of ConA Sepharose Suspension (Merck, Darmstadt, Germany) were washed 5 times with 1 ml Binding Buffer (20 mM Tris (pH 7.4), 150 mM NaCl, 1 mM $MnCl_2$, 1 mM $CaCl_2$). Meanwhile, 2 mg samples after tryptic digestion were redissolved in 1 ml Binding Buffer by short sonication and 30 min incubation at RT with gentle shaking and added to prepared ConA suspension. The sample was then incubated overnight on a rotator at 4 °C. Next day the beads were washed 5 times with 1 ml Binding Buffer and membrane glycopeptides were eluted 3 times with 0.5 ml Binding Buffer + 0.5 M methyl-mannopyranoside and incubation for 1h on a rotator at 4 °C.

2.6 *Anion exchange chromatography*

Glycopeptides after Stage Tipping were resuspended in 1 ml 20 mM Tris pH 8.0 by sonication and 30 min incubation at RT with gentle shaking. Glycopeptides were further fractionated on anion exchange column: MiniQ 4.6/50 PE (Merck, Darmstadt, Germany) using ÄKTA purifier (GE Healthcare, Chicago, IL, USA). Sample was fractionated over 0 -1 M NaCl gradient (flow 0.5 ml/min): 0-0.3 M in 12 ml, 03. -0.5 M in 20 ml and 0.5 – 1.0 M in 20 ml.

2.7 *Reverse Phase chromatography*

Elution from glycopeptide enrichment was mix with 3.5 ml solvent A: 2% acetonitrile and 0.1% formic acid. Glycopeptides were further fractionated on reverse phase column: Resource RPC (1 ml; Merck, Darmstadt, Germany) using ÄKTA purifier (GE Healthcare, Chicago, IL, USA) over a stepped linear gradient of solvent A and solvent B (99.9% acetonitrile and 0.1% formic acid) in the following sequence from 0 to 4% B

in 2 ml, from 4 to 40% D in 40 ml, from 40 to 60% D in 4 ml, 6 ml at 85% B, from 85 to 0% B in 2 ml, and 8 ml at 0% B.

2.8 *SDS-PAGE*

SDS-PAGE was performed using Novex™ 16% Tricine gels (Thermo Fisher Scientific, Karlsruhe, Germany). Gels were stained with Coomassie (Instant Blue™, Expedeon, Heidelberg, Germany) or Silver Stain using Pierce™ Silver Staining Kit (Thermo Fisher Scientific) according to manufacturers' instructions.

2.9 *Peptide Standard and RNase B glycopeptide analysis*

0.5 mg of RNase B from bovine pancreas (Merck) were digested following in-solution digestion in NH_4HCO_3 – buffer (Supplementary Information Section 2.1) and glycopeptide enrichment (Materials and Methods Section 4.4). 2 replicates of 50 μg of Peptide Standard (MS QCAL Peptide Mix, Merck) and RNase B glycopeptides were fractionated on Size Exclusion column as described in Materials and Methods Section 4.5.

Fractions collected from RNase B glycopeptide fractionation underwent StageTipping as described in Materials and Methods Section 4.3. and resuspended in 5 μl of 0.1% TFA. 1 μl from each fraction was spotted on MALDI target plate followed by 1 μl of α-cyano-4-hydroxy-cinnamic (HCCA, Bruker Daltonik, Bremen, Germany)) matrix in acetonitrile:0.1% TFA (50:50). Fractions collected from Peptide Standard were desalted using ZipTip (C18, Merck) according to manufacturer's advise and were eluted with 2 μl HCCA matrix in acetonitrile:0.1%TFA (50:50) directly on MALDI target plate. Spotted samples were dried at ambient temperature prior to mass spectrometric analysis. MALDI mass spectra were collected on an rapifleX™ Tissue Typer instrument (Bruker Daltonik). Spectra were processed with ProteoWizard [4] and Mmass [5] software. Peaks were identified with peak-to-noise ratio of 3. Each fraction was analysed separately and elution of glycopeptide and peptide standards were compared using the methodology for glycosite profiling described in Materials and Methods section 4.9.

References

1. Wiśniewski, J.R.; Zougman, A.; Nagaraj, N.; Mann, M. Universal sample preparation method for proteome analysis. *Nat Methods* **2009**, *6*, 359-362, doi:10.1038/nmeth.1322.
2. Lee, Y.C.; Block, G.; Chen, H.; Folch-Puy, E.; Foronjy, R.; Jalili, R.; Jendresen, C.B.; Kimura, M.; Kraft, E.; Lindemose, S., et al. One-step isolation of plasma membrane proteins using magnetic beads with immobilized concanavalin A. *Protein Expr Purif* **2008**, *62*, 223-229, doi:10.1016/j.pep.2008.08.003.
3. Michalak, M.; Warnken, U.; Schnölzer, M.; Gabius, H.J.; Kopitz, J. Detection of malignancy-associated phosphoproteome changes in human colorectal cancer induced by cell surface binding of growth-inhibitory galectin-4. *IUBMB Life* **2019**, *71*, 364-375, doi:10.1002/iub.1987.
4. Chambers, M.C.; Maclean, B.; Burke, R.; Amodei, D.; Ruderman, D.L.; Neumann, S.; Gatto, L.; Fischer, B.; Pratt, B.; Egertson, J., et al. A cross-platform toolkit for mass spectrometry and proteomics. *Nat. Biotechnol.* **2012**, *30*, 918-920, doi:10.1038/nbt.2377.
5. Strohalm, M.; Hassman, M.; Kosata, B.; Kodícek, M. mMass data miner: an open source alternative for mass spectrometric data analysis. *Rapid Commun. Mass Spectrom.* **2008**, *22*, 905-908, doi:10.1002/rcm.3444.
